# The epidemic of alignment classifications in total knee arthroplasty forgives the kinematic of the human knee

**DOI:** 10.1002/jeo2.70052

**Published:** 2024-10-24

**Authors:** Pier Francesco Indelli

**Affiliations:** ^1^ Südtiroler Sanitätsbetrieb Brixen Italy; ^2^ Institute of Biomechanics Paracelsus Medical University Salzburg Austria; ^3^ Paolo Aglietti Gait Lab, CESAT Azienda Sanitaria Toscana Centro Fucecchio Italy; ^4^ The Breyer Center for Overseas Studies Stanford University in Florence Florence Italy; ^5^ Ortho Team Research ETS Florence Italy; ^6^ European Society of Sports Traumatology, Knee Surgery Arthroscopy (ESSKA) Basic Science Committee Luxembourg

**Abbreviations**
AMA: adjusted mechanical alignment
AR: augmented reality
CAOS: computer‐assisted orthopedic surgery
CPAK: coronal plane alignment of the knee
dHKA: dynamic hip–knee–ankle
HKA: hip–knee–ankle
KA: kinematic alignment
PROMs: patient‐reported outcome measures
TKA: total knee arthroplasty

The constitutional limb alignment has been historically described as varus, neutral, or valgus while, in modern times, it has been defined as the hip–knee–ankle angle (HKA). This alignment measurement represents the angle between the mechanical axes of the femur and the tibia, and it is routinely measured on a static, full‐length lower‐limb radiograph. Arthroplasty surgeons, for more than 30 years [11], had the intraoperative goal of reproducing a neutral HKA during the total knee arthroplasty (TKA) procedure, disregarding individual morphological variations for reliability and simplicity.

The recent, growing interest in individualized alignment techniques in TKA has created a push in the development of multiple radiological classifications to categorize the coronal alignment of knee phenotypes during weightbearing. Lin et al. [12] were among the first to propose a classification system with 27 possible phenotypes but only five of them were originally considered clinically relevant. Later, Hirschmann et al. [6] introduced the concept of the functional knee phenotype, with 125 possible phenotypes, 43 of which were considered clinically relevant. In 2021 MacDessi et al. [13] proposed the Coronal Plane Alignment of the Knee (CPAK) Classification, defining nine CPAK phenotypes. These classifications [6, 12, 13] combined several variables, including the mechanical limb alignment, the proximal tibial angle, the distal femoral angle, the joint line obliquity, and the arithmetic HKA. The common denominator of all these classification systems is characterized by the fact that all measurements were taken on double‐leg, weightbearing, long‐leg radiographs which represent a “static” modality. These multiple phenotypes have become the targets of multiple surgical techniques, recently developed to support surgeons in the intraoperative decision‐making process: the adjusted mechanical alignment (AMA) [7], the anatomical alignment [9], the kinematic alignment (KA) [8], the restricted KA [21], the inverse KA [22] and, finally, the functional alignment [16] were all designed to match the patient's constitutional alignment as measured on the preoperative standing films.

Interestingly, this massive research effort did not take into consideration that the HKA axis varies significantly during the gait cycle: the hypothesis that the standing coronal alignment remains constant during the gait cycle, especially at the first (mid‐stance phase of the gait cycle) and second knee flexion peaks (mid‐swing phase of the gait cycle), remains highly controversial [16]. Miller et al. [14] showed that the dynamic loading on the tibial plateau is affected by limb position, muscle contraction, soft‐tissue stability, and walking speed, hypothesizing that the standing coronal alignment does not predict the dynamic alignment of the limb and the dynamic adduction moment at the knee. Similarly, Riviere et al. [19] highlighted the limited value of standing coronal alignment after TKA to anticipate dynamic frontal parameters, either the alignment of the knee or the adduction moment. Because of this knowledge, multiple authors, including from the current author Institution [1, 2, 3], supported the concept of a dynamic HKA (dHKA), which ultimately measures the coronal alignment of the knee throughout the gait cycle, as an alternative measure to the static HKA as measured on standing, long‐leg films. Duffel et al. [3] showed that the dHKA could be in significantly greater varus during the gait cycle than the static HKA. Clément et al. [2], in a study on 90 healthy individuals, showed a mean variation of dHKA during gait of almost 11°: moreover, they found a low to moderate correlation between static HKA and dHKA values for varus knees and no correlation for valgus knees; interestingly, the largest variation occurred during the swing phase of the gait cycle. Similarly, in a gait analysis study following TKA procedures, Orishimo et al. [15] did not find any correlation between static or dynamic coronal alignment during the entire gait cycle.

It is the current's author opinion that the literature on knee kinematics supports the limited value of weightbearing radiographic measures of coronal alignment for predicting dynamic measures of alignment during gait. The increasing effort to postoperatively reproduce the constitutional lower limb alignment according to one of the multiple phenotype classifications [6, 12, 13] may miss the real target which is a clinical one: reducing patients' symptoms, which historically occur mainly in the dynamic phases of the gait cycle. At the current time, instability, and infection (not malalignment) represent the most cited causes for TKA failure [10]. It has been shown that unstable TKAs differ from stable TKAs in kinematic parameters both in the early stance phase (greater knee flexion excursion) as well as in the early/mid‐swing phase (condylar posterior translation followed by an additional anterior translation) of the gait cycle [4, 18].

Having the possibility of defining the dHKA and the intercompartmental gap symmetry/asymmetry during those crucial gait cycle phases has a major theoretical advantage in avoiding instability following TKA. Enabling technologies represent an extremely valuable tool for TKA surgeons [17] but their use should not be limited to targeting the knee phenotype as determined by measuring the static HKA: augmented reality, computer‐assisted navigation, and robotic systems are currently able to give real‐time feedback on multiple intra‐operative parameters, including dHKA (Figure 1), which has been shown to correlate better with postoperative kinematic, implant survival and Patients Reported Outcome Measures (PROMs) [5] respect to the static HKA [20].

**Figure 1:**
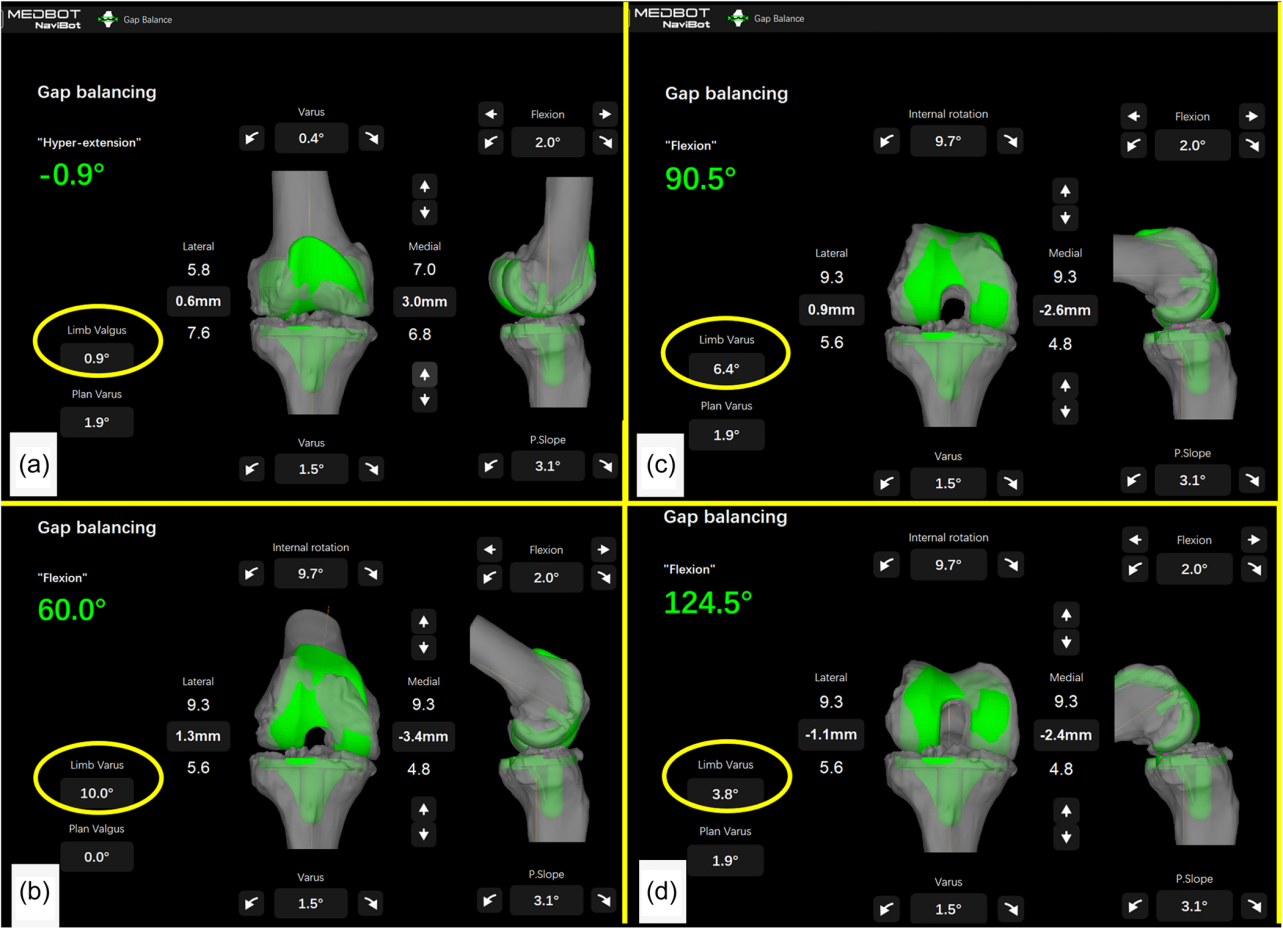
Robotic assisted right knee TKA. Intraoperative evaluation of the knee ROM from full extension (a) to maximum flexion (d): the alignment of the knee is also intraoperatively determined. (a) Knee in full extension (slight hyperextension): 0.9° valgus; (b) knee flexed at 60°: 10° varus; (c) Knee flexed at 90.5°: 6.4° varus; (d) Knee flexed at 124.5°: 3.8° varus. The evaluation has been performed without applying any adduction/abduction moment to the hip or varus/valgus stress to the knee (Skywalker Robotic System, Medbot Navi‐Bot). The dynamicity of the HKA is intraoperatively noted. HKA, hip‐knee‐ankle axis; ROM, range of motion; TKA, total knee arthroplasty.

In conclusion, in a time of proliferation and investigation of individual, knee morphology radiological classifications, there is a need for studies showing the benefit of reproducing a personalized, dynamic HKA alignment after TKA in terms of implant survivorship as well as the reproduction of close‐to‐normal knee kinematics. Time will tell if a more personalized, dynamic approach for TKA alignment will lead to better clinical outcomes and fewer dissatisfied patients.

## CONFLICT OF INTEREST STATEMENT

The author declares no conflict of interest.

## ETHICS STATEMENT

The author has nothing to report.
